# Bufalin Reverses HGF-Induced Resistance to EGFR-TKIs in EGFR Mutant Lung Cancer Cells via Blockage of Met/PI3k/Akt Pathway and Induction of Apoptosis

**DOI:** 10.1155/2013/243859

**Published:** 2013-02-28

**Authors:** Xiao-Hong Kang, Zhen-Ye Xu, Ya-Bin Gong, Li-fang Wang, Zhong-Qi Wang, Ling Xu, Fei Cao, Ming-juan Liao

**Affiliations:** ^1^Department of Clinical Oncology, Long Hua Hospital, Shanghai University of Traditional Chinese Medicine, Shanghai 200032, China; ^2^Department of Clinical Oncology, Ping Ding Shan First People's Hospital, Henan 467000, China; ^3^Department of Infection, Ping Ding Shan First People's Hospital, Henan 467000, China

## Abstract

The epidermal growth factor receptor tyrosine kinase inhibitors (EGFR-TKIs), such as gefitinib and erlotinib, have shown promising therapeutic efficacy in nonsmall cell lung cancer (NSCLC) patients harboring epidermal growth factor receptor- (EGFR-) activating mutation. However, the inevitable recurrence resulting from acquired resistance has limited the clinical improvement in therapy outcomes. Many studies demonstrate that hepatocyte growth factor- (HGF-) Met axis plays an important role in tumor progression and drug sensitivity. HGF may induce resistance to EGFR-TKIs in EGFR mutant lung cancer cells by Met/PI3K/Akt signaling. The purpose of this study was to determine whether bufalin, a major bioactive component of Venenum Bufonis, could reverse HGF-induced resistance to reversible and irreversible EGFR-TKIs in mutant lung cancer cells PC-9, HCC827, and H1975. Our studies showed that bufalin could reverse resistance to reversible and irreversible EGFR-TKIs induced by exogenous HGF in EGFR mutant lung cancer cells by inhibiting the Met/PI3K/Akt pathway and inducing death signaling. These results suggested that bufalin might have a potential to overcome HGF-induced resistance to molecular-targeted drugs for lung cancer.

## 1. Introduction

Lung cancer is the leading cause of cancer-related death in the world. Nonsmall cell lung cancer (NSCLC) accounts for nearly 80% of lung cancer cases. Recent intensive molecular analyses of lung cancers have identified several molecular aberrations occurring in protooncogenes that are mutually exclusive to each other [1]. Notably, the proliferation and survival of lung cancers harboring one of these molecular aberrations often depend on aberrant signaling from the mutated oncogene, the so-called oncogene addiction phenomenon [2]. Epidermal growth factor receptor (EGFR) is expressed in up to 80%–90% of NSCLC [3] and plays a vital role in the pathogenesis. Lung cancers that depend on mutated EGFR constitute one of the biggest lung cancer subsets characterized by molecular aberrations, accounting for ~50% in East Asians and ~15% in Caucasians [4]. Because EGFR-mutated lung cancers are dependent on mutant EGFR, the EGFR tyrosine kinase inhibitors (TKIs) show promising therapeutic efficacy in patients with EGFR-activating mutations, such as exon 19 deletions and L858R point mutations [5]. However, almost all patients develop acquired resistance to EGFR-TKIs within one year [6], thus limiting the outcome improvement in patients. Among the molecular mechanisms of this acquired resistance to EGFR-TKIs are EGFR T790M secondary mutation and bypass signaling caused by Met amplification or hepatocyte growth factor (HGF) overexpression [7–9]. In addition, PIK3CA mutations and transformation to SCLC have also been found to contribute to EGFR-TKIs resistance in a subpopulation of tumors [10]. Many studies have reported recently that HGF overexpression was involved not only in the acquired resistance but also in the intrinsic resistance to EGFR-TKIs. It was found that HGF induced resistance to reversible, irreversible, and even mutant-selective EGFR-TKIs by restoring MetGab1/PI3K/Akt pathway [11–14], indicating that HGF is an important therapeutic target for overcoming tumor resistance to EGFR-TKIs.

Bufalin is a major bioactive component of Venenum Bufonis, a traditional Chinese medicine obtained from the skin and parotid venom glands of toads [15–17], and has been found to induce cell apoptosis in various types of cancer cells, including hepatocellular carcinoma [18, 19], colon cancer [20], leukemia [21], gastric cancer [22], prostate cancer [23], and malignant melanoma [24]. Recently, some reports have shown that bufalin inhibited proliferation of human lung cancer cells by blocking PI3k/Akt pathway [25, 26]. Based on the concept that inhibition of PI3K/AKT pathway may effectively overcome HGF-induced resistance to gefitinib, we hypothesized that bufalin could reverse EGFR-TKIs resistance induced by HGF in EGFR mutant lung cancer. We, therefore, assessed whether bufalin combined with EGFR-TKIs could overcome HGF-induced resistance to EGFR-TKIs *in vitro*.

## 2. Materials and Methods

### 2.1. Cell Culture

The EGFR mutant human lung adenocarcinoma cell lines PC-9, HCC827, and H1975 were purchased from American Type Culture Collection (ATCC) and maintained in RPMI-1640 medium supplemented with 10% FBS, 100 units/mL penicillin, 100 *μ*g/mL streptomycin, and 2 mM L-glutamine at 37°C in 100% humidity, 5% CO_2_, and 95% air. Cell line characterization and authentication were carried out by the ATCC Molecular Authentication Center, using cytochrome c oxidase subunit I (COI) for interspecies identification and short tandem repeat (STR) analysis (DNA fingerprinting) for intraspecies identification. All cells were passaged for less than 3 months before renewal from frozen, early-passage stocks. 

### 2.2. Reagents

**Figure 1:**
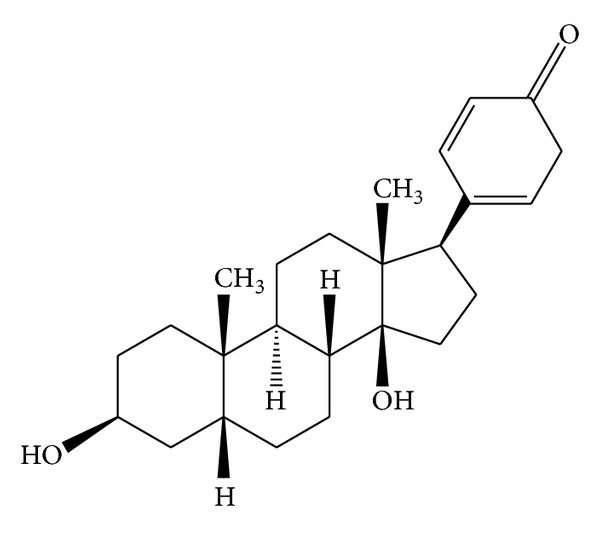
Chemical structure of bufalin.

Bufalin was obtained from the Sigma-Aldrich. The reversible EGFR-TKI, gefitinib, the irreversible EGFR-TKI, BIBW2992 (afatinib), and Met inhibitors SU11274 were purchased from Selleck. Human recombinant HGF was obtained from Peprotech. The chemical structure of bufalin was shown in Figure 1.

### 2.3. Cell Proliferation Assay

Cell proliferation was measured by the MTT [3-(4,5-dimethylthiazol-2-yl)-2,5-diphenyl tetrazolium] dye reduction method [27]. Tumor cells were plated at a density of 3 × 10^3^ cells/100 *μ*L/well into 96-well plates in RPMI-1640 medium with 10% FBS. After 24 h incubation, various reagents were added to each well, and the cells were incubated for an additional 72 h, and then followed by the addition of 20 *μ*L of MTT solution (5 mg/mL; Sigma) to each well and further incubation for 4 h. The media containing MTT solution was removed, and the dark blue crystals were dissolved with 150 *μ*L of dimethyl sulfoxide (DMSO). The optical density (OD) of each well was measured with a 96-well microplate reader (BIO-RAD) at test and reference wavelengths of 550 and 630 nm, respectively. The percentage of growth is shown relative to the untreated controls. Each reagent and concentration was tested at least in triplicate during each experiment, and each experiment was conducted at least 3 times.

### 2.4. Cell Apoptosis Assay

Cell apoptosis was detected by an Annexin V-FITC/PI Apoptosis Detection kit I (BD Biosciences) in accordance with the manufacturer's protocols. Cells were seeded into 6-well plate at the density of 1 × 10^5^/well. After incubation for 24 h at 37°C, gefitinib (1 *μ*M), bufalin (20 nM), and bufalin plus gefitinib, with HGF (30 ng/mL) were added to the 6-well plate and incubated for another 24 h. Cells were collected and washed twice with PBS and then resuspended, and aliquots of 1 × 10^5^ cells were transferred into new 5 mL culture tubes in 100 *μ*L of 1 × binding buffer. Then, 5 *μ*L of Annexin V-FITC and 5 *μ*L of propidium iodide were added to the resuspended cells. After incubation at room temperature for 15 min in the dark, 400 *μ*L of binding buffer were added to the resuspended cells. Flow cytometry (Becton Dikinson, USA) was used to assess the apoptotic cells. The quantitation of apoptotic cells was calculated by CellQuest software.

### 2.5. Antibodies and Western Blotting

Lung cancer cells at a density of 1 × 10^5^/well were seeded into 6-well plates and incubated for 24 h at 37°C, and then treated with gefitinib (1 *μ*M) and/or bufalin (20 nM) in the presence or absence of HGF for another 24 h. Culture cells were washed twice with ice-cold PBS and lysed RIPA buffer containing phosphatase inhibitor cocktail and proteinase inhibitor cocktail (Roche, UK), and the protein concentrations were determined using a BCA Protein Assay Kit (Pierce, Rockford, 1L, USA). 40 *μ*g of total proteins was subjected to SDS-PAGE under reducing conditions and transferred to PVDF membranes (Millipore, Bedford, MA, USA). The membranes were blocked with 5% nonfat milk for 2 h at room temperature followed by overnight incubation at 4°C with the following antibodies: anti-EGFR, anti-p-EGFR(Y1068), anti-MET, antiphospho-MET (Y1234/Y1235) (1 : 2000 dilution, Epitomics), anti-PI3k p85, or phospho-PI3k p85 (Tyr458)/p55 (Tyr199), anti-AKT, or phospho-AKT (Ser473), anticleaved caspase-3, anticleaved caspase-9, and anticleaved PARP antiantibodies (1 : 1000 dilution, Cell Signaling Technology). After washing 3 times, the membranes were incubated for 1 h at room temperature with species-specific horseradish peroxidase-conjugated secondary antibodies. The intensity of blot signals was quantified using ImageQuant TL analysis software (General Electric, UK). Each experiment was performed at least three times independently.

### 2.6. Statistical Analysis

The quantitative data are shown as mean ± SD. All statistical analyses were performed using SPSS Version 18.0 (Chicago, IL, USA), and differences were analyzed by one-way ANOVA. *P* < 0.05 was considered significant.

## 3. Results

### 3.1. Bufalin Overcomes the Resistance to Reversible EGFR-TKIs Induced by HGF via Inhibition of Met/PI3K/Akt Pathway

**Figure 2:**
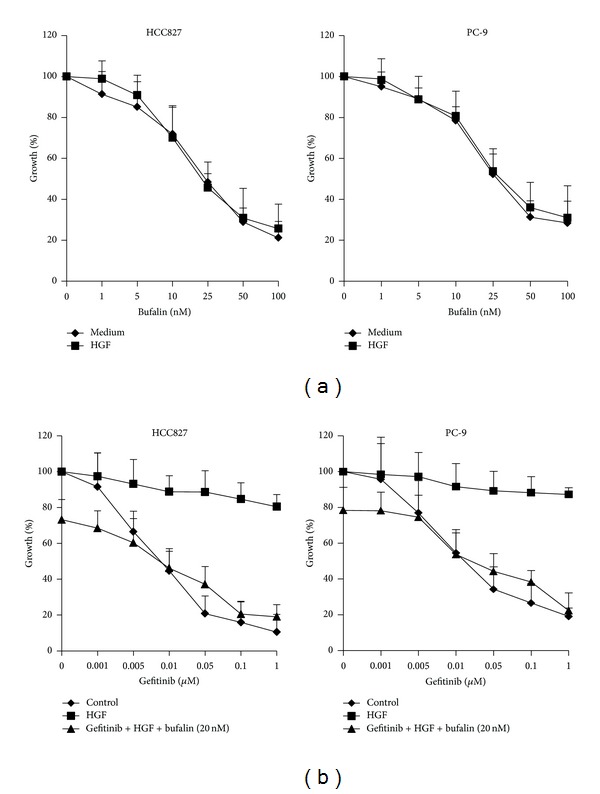
Bufalin combined with gefitinib overcomes HGF-triggered gefitinib resistance. (a) HCC827 and PC-9 cells were treated with various concentrations of bufalin, with or without HGF (30 ng) for 72 h. (b) HCC827 and PC-9 cells were treated with various concentrations of gefitinib, with or without bufalin (20 *μ*M) for 72 h, in the presence or absence of HGF (30 ng). Cell growth was determined by MTT assay. Error bars indicate SD.

PC-9 and HCC827, the EGFR mutant human lung cancer cell lines with EGFR exon 19 deletion, were highly sensitive to gefitinib [28]. Whereas exogenous addition of HGF to both the two types of cells led to resistance to gefitinib as others reported previously [11, 29–31]. Continuous exposure to bufalin for 72 h inhibited the proliferation of PC-9 and HCC827 cells in a concentration-dependent manner, even in the presence of HGF. We then assessed the effects of combined therapy with bufalin and gefitinib on PC-9 and HCC827 cells in the presence of HGF. Although HGF induced resistance to gefitinib in both cell lines, bufalin combined with gefitinib further suppressed the proliferation of PC-9 and HCC827 cells (Figure 2). These findings indicated that bufalin could reverse exogenous HGF-induced resistance to reversible EGFR-TKIs *in vitro*.

Many studies recently reported that HGF induced resistance to reversible EGFT-TKIs in EGFR mutant lung cancer cells by activating Met and the downstream phosphoinositide 3-kinase (PI3K)/Akt pathway [11–14, 31, 32]. We then examined the EGFR and Met/PI3K/Akt signal transduction status, using western blotting, to explore the molecular mechanism by which bufalin combined with gefitinib showed greater antiproliferative effect on HCC827 cells, which were exposed to HGF. We found that exogenous HGF stimulated the phosphorylation of Met and thereby activated the downstream molecules PI3K and Akt. Gefitinib inhibited the phosphorylation of EGFR, but failed to inhibit the phosphorylation of PI3K and Akt in HCC827 cells exposed to HGF. Under the same experimental condition, bufalin did not affect the expression of total EGFR, Met, PI3K, and Akt, but inhibited the phosphorylation of EGFR slightly and the phosphorylation of Met, PI3K, and Akt considerably. In addition, bufalin combined with gefitinib markedly inhibited the phosphorylation of EGFR, Met, PI3K, and Akt (Figure 4). These results suggested that bufalin reversed HGF-induced gefitinib resistance by inhibiting the MET/PI3K/AKT pathway.

To further confirm that bufalin overcame this resistance by blocking Met/PI3K/Akt signal pathway, we then inhibited Met by Met-TKIs SU11274 in these gefitinib resistant cells. Treatment with SU11274 (5 *μ*M) plus gefitinib successfully reversed HGF-induced resistance to gefitinib by inhibiting the phosphorylation of both Met and Akt (Figure 4). These evidence further supported that bufalin sensitized these cells to gefitinib by inhibiting the Met/PI3K/Akt pathway.

### 3.2. Bufalin Overcomes the Resistance to Irreversible EGFR-TKIs Induced by HGF via Inhibition of Met/PI3K/Akt Pathway

**Figure 3:**
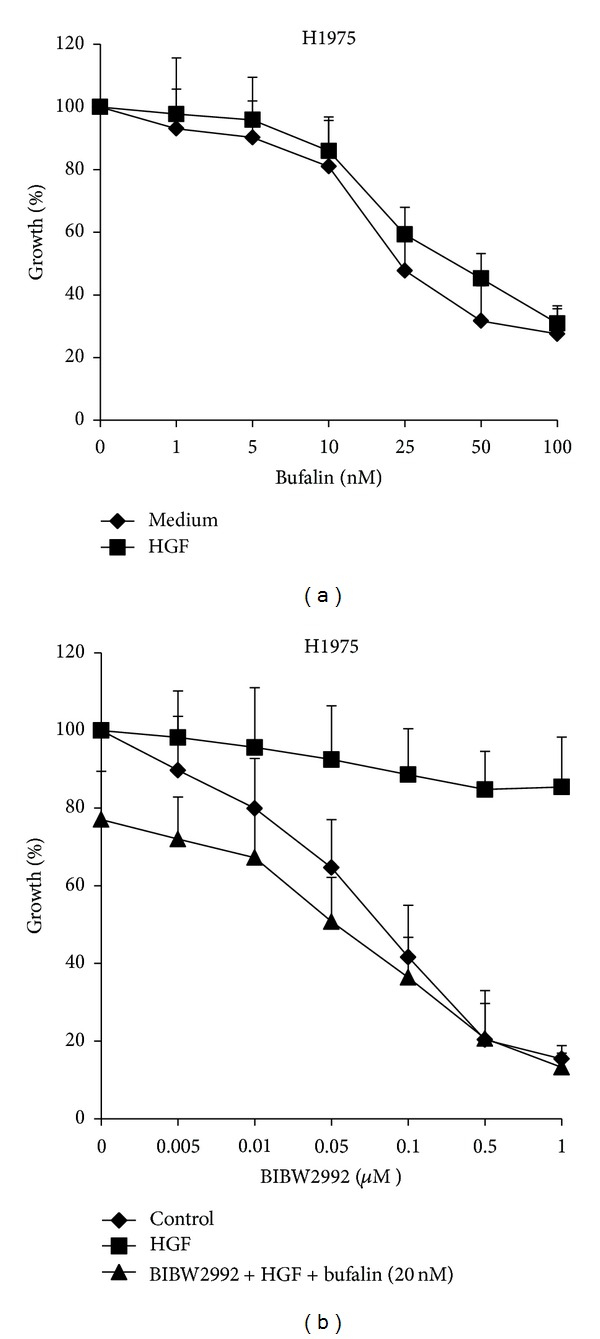
Bufalin combined with BIBW2992 overcomes HGF-triggered resistance to irreversible EGFR-TKIs in H1975 cells. (a) H1975 cells were treated with various concentrations of bufalin, with or without HGF (30 ng) for 72 h. (b) H1975 cells were treated with various concentrations of BIBW2992, with or without bufalin (20 *μ*M) for 72 h, in the presence or absence of HGF (30 ng). Cell growth was determined by MTT assay. Error bars indicate SD.

H1975 cancer cell line with mutations in EGFR exons 21 (L858R) and 20 (T790M) was refractory to reversible EGFR-TKIs, gefitinib, and erlotinib [33], but was sensitive to irreversible EGFR-TKIs, such as BIBW2992 (afatinib). Exogenous HGF triggered resistance in H1975 to BIBW2992, as others described previously [31, 32]. Interestingly, continuous exposure to bufalin for 72 h inhibited the proliferation of H1975 cells in a dose-dependent manner, regardless of presence of HGF. In addition, bufalin combined with BIBW2992 markedly suppressed the proliferation of H1975 in presence of HGF, suggesting that bufalin had potential to reverse HGF-induced resistance to irreversible EGFR-TKIs (Figure 3).

**Figure 4:**
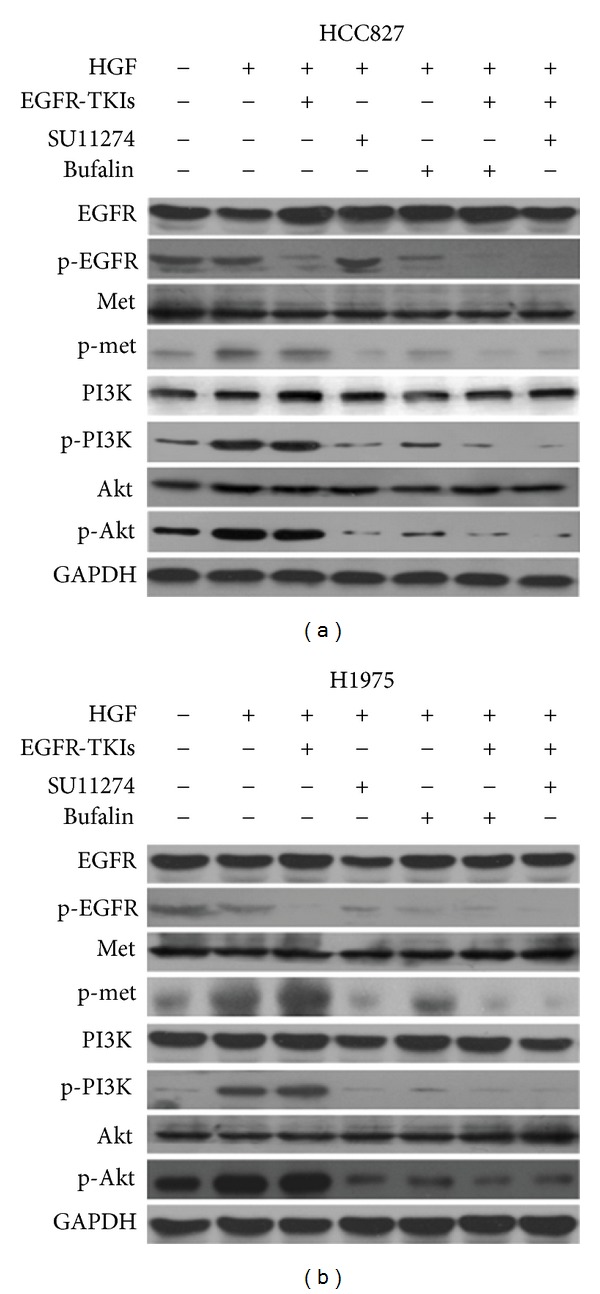
Bufalin combined with EGFR-TKIs overcomes resistance to EGFR-TKIs induced by HGF via inhibition of Met/PI3k/Akt pathway. (a) HCC827 cells were treated with bufalin (20 nM), SU11274 (5 *μ*M), and/or gefitinib (1 *μ*M) for 24 h, in the presence or absence of HGF (30 ng). (b) H1975 cells were treated with bufalin (20 nM), SU11274 (5 *μ*M), and/or BIBW2992 (0.1 *μ*M) for 24 h, in the presence or absence of HGF (30 ng). Then cells were lyzed, and the associated proteins were detected by western blotting.

Using western blotting analyses, we examined the effects of bufalin on signal transduction in H1975 cells in the presence or absence of HGF. The results showed that bufalin inhibited the phosphorylation of EGFR and Met and thereby the downstream molecules PI3K and Akt at various levels in H1975 cells in the presence of HGF. Although BIBW2992 inhibited EGFR phosphorylation, the phosphorylation of PI3K and Akt was not inhibited, and the phosphorylation of Met was even enhanced in H1975 cells in the presence of HGF, indicating that HGF activated the Met/PI3K/Akt pathway and resulted in resistance to irreversible EGFR-TKIs. Combination of bufalin and BIBW2992 further inhibited the phosphorylation of EGFR, MET, and the downstream PI3K and Akt. We further confirmed that bufalin overcame irreversible EGFR-TKIs resistance by inhibiting the phosphorylation of MET and the downstream PI3K/AKT with SU11274. Combination treatment of SU11274 and BIBW2992 totally abrogated this HGF-triggered resistance by suppressing the phosphorylation of both Met and Akt (Figure 4). These findings suggested that bufalin overcame HGF-triggered irreversible EGFR-TKIs resistance by inhibiting the Met/PI3K/Akt pathway.

### 3.3. Bufalin Further Restored EGFR-TKIs Sensitivity in the Presence of HGF by Activating Apoptosis Signaling

**Figure 5:**
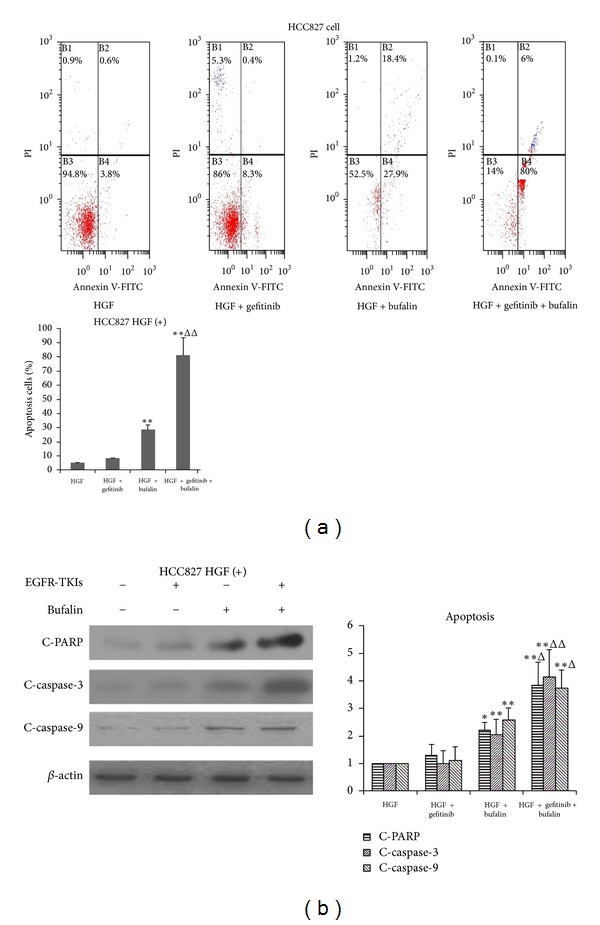
Bufalin combined with gefitinib overcomes resistance to gefitinib induced by HGF via activation of apoptosis signaling. (a) HCC827 cells were treated with gefitinib (1 *μ*M) and/or bufalin (20 nM), in the presence of HGF (30 ng) for 24 h, and then stained with Annexin V/PI and detected by flow cytometry. (b) HCC827 cells were treated with gefitinib (1 *μ*M) and/or bufalin (20 nM), in the presence of HGF (30 ng) for 24 h. Then cells were lyzed, and the associated proteins were detected by western blotting. Each bar represented mean ± SD. *P < 0.05 and **P < 0.01 compared with gefitinib, ^Δ^ *P* < 0.05 and ^ΔΔ^ *P* < 0.01 compared with bufalin.

The evasion of apoptosis is a hallmark of cancer and one of the reasons for drug resistance. Many studies have identified that bufalin induced apoptosis of many cancer cells. We then investigated the effects of bufalin plus EGFR-TKIs on the cell apoptosis using flow-cytometry analyses with Annexin V. The findings showed that gefitinib failed to induce obvious apoptosis in HCC827 cells in the presence of HGF. In contrast, bufalin induced apoptosis of HCC827 cells in the presence of HGF. Moreover, combination of bufalin and EGFR-TKIs markedly induced apoptosis of HGF-treated HCC827 cells, indicating that bufalin potentiated the antineoplastic activity of EGFR-TKIs by apoptosis induction (Figure 5).

To further confirm these observations, we examined the expression of various apoptosis-related proteins in HCC827 cells by western blotting assays. In the presence of HGF, gefitinib did not affect the expressions of cleaved-PARP, cleaved-caspase-3, and cleaved-caspase-9 in HCC827 cells. In contrast, bufalin stimulated the expression of cleaved-PARP, cleaved-caspase-3 and cleaved-caspase-9 in HCC827 cells. Importantly, treatment with bufalin with gefitinib markedly increased the abundance of cleaved-PARP, cleaved-caspase-3, and cleaved-caspase-9 (Figure 5). These findings indicated that bufalin further restored EGFR-TKIs sensitivity by activating apoptotic signaling in the presence of HGF.

## 4. Discussion

Lung cancers that harbor somatic activating mutations in the gene for EGFR depend on mutant EGFR for their proliferation and survival; therefore, lung cancer patients with EGFR mutation often dramatically respond to orally available EGFR-TKIs. Recent clinical trials have shown that first-line treatment for EGFR-mutated lung cancers with EGFR-TKIs induced much higher response rates and longer progression-free survival than platinum-based chemotherapies [34–37]. Almost all patients, however, develop acquired resistance to EGFR-TKIs after varying periods of time [6]. In the analyses of clinical specimens obtained from patients who acquired resistance to EGFR-TKIs, high expression of HGF was identified in 61% patients, overlapping with T790M mutation and MET amplification. HGF expression was significantly higher in tumors with acquired resistance than in pretreatment sensitive tumors [38], and has been associated with poor prognosis in patients resected for NSCLC [39]. In addition, many studies have shown the important roles of HGF in sensitivity to molecular-targeted drugs. The concentrations of HGF in peripheral blood were found to be inversely correlated with clinical responses to EGFR-TKIs in both EGFR mutant and wild-type lung cancer [40]. HGF frequently detected in EGFR-TKIs resistant tumors with EGFR-T790M second mutation and may induce the resistance to irreversible EGFR-TKIs [13]. Moreover, HGF was also found to induce resistance to sunitinib [41], ATE684 (a selective ALK inhibitor) [42], vemurafenib (the BRAF inhibitor, in BRAF-mutant melanoma) [43, 44], and lapatinib (a dual HER2 and EGFR tyrosine kinase inhibitor) [45]. These findings indicated that reducing the expression of HGF or blockage of HGF-Met signaling were promising strategies for overcoming drug resistance in cancer.

Several reports have shown that bufalin inhibited cell proliferation and induced cell apoptosis via the inhibition of PI3K/AKT pathway in many cancer cells [22, 25, 26]. However, there is no report showing that bufalin could overcome HGF-induced EGFR-TKIs resistance in EGFR mutant lung cancer cells when combined with gefitinib or afatinib. We have shown here for the first time that bufalin restored EGFR-TKIs sensitivity in resistant lung cancer cells via inhibition of Met/PI3K/Akt and activation of death signaling. 

HGF could induce resistance to EGFR-TKIs via Met/PI3K/AKT activation, indicating that to overcome resistance to EGFR-TKIs induced by HGF-triggered activation of Met/PI3K/AKT pathway in mutant lung cancer, it was necessary to double blockade EGFR and HGF-Met signaling pathways. 

We showed here that bufalin combined with EGFR-TKIs further inhibited the proliferation of PC-9, HCC827, and H1975 cells in the presence of HGF. Although bufalin inhibited EGFR phosphorylation slightly compared with control, it suppressed the phosphorylation of both Met and PI3k/Akt in HCC827 and H1975 cells. Combination of bufalin and EGFR-TKIs successfully inhibited mutant EGFR and HGF-Met signaling pathway. These results suggested that bufalin reversed HGF-induced EGFR-TKIs resistance in EGFR mutant lung cancer cells by inhibiting the phosphorylation of Met and the downstream PI3K/Akt, and Met could be one of the therapeutic targets of bufalin. Further experiments are now ongoing in our laboratory to confirm whether Met is the target of bufalin.

In addition, resistance to apoptosis was an important hallmark of tumor cells [46]. Activation of antiapoptosis signaling pathway was involved in resistance to targeted drugs [47]. Flow cytometry analyses have shown that the combination of bufalin and gefitinib induced apoptosis of HCC827 cells, although HGF rendered EGFR mutant cancer cells resistance to apoptosis induced by EGFR-TKIs. In apoptosis signaling pathway, cleaved-caspase-9, the initiator caspase, activates the effector caspase-3. In turn, the activation of caspase-3 cleaves a variety of substrates, such as poly-(ADP-ribose) polymerase (PARP) and eventually dismantled cells [48, 49]. Therefore, cleaved-caspases-3 and cleaved-PARP are the hallmarks and indicator of apoptosis. We then tested the expression of cleaved-caspase-3 and -9 and cleaved-PARP in HCC827 cells in the presence of HGF. Treatment of bufalin plus gefitinib markedly increased the levels of cleaved-caspase-3 and -9 and cleaved-PARP, indicating that activation of death signaling could be associated with bufalin reversal of the resistance to EGFR-TKIs induced by HGF. However, apoptosis signing may be induced by the death receptor-dependent pathway (extrinsic pathway) or the mitochondria-dependent pathway (intrinsic pathway) [50, 51]. We, thus, need to demonstrate the precise mechanisms by which bufalin overcame HGF-triggered resistance to apoptosis in EGFR-mutant lung cancer.

In conclusion, we showed here that bufalin could reverse HGF-induced resistance to EGFR-TKIs by inhibiting Met/PI3K/Akt pathway and activating death signaling in EGFR mutant lung cancer cells. We have presented the evidence that the combination of EGFR-TKIs and antitumor active ingredients from natural resources would be a promising strategy to overcome EGFR-TKIs resistance.
